# Fleas of wild carnivores in Romania: diversity, distribution, and host-associations

**DOI:** 10.1186/s13071-024-06235-3

**Published:** 2024-03-21

**Authors:** Georgiana Deak, Angela Monica Ionică, Áron Péter, Attila David Sándor, Ioana Adriana Matei, Gianluca D’Amico, Emmanuel Liénard, Călin Mircea Gherman, Andrei Daniel Mihalca, Emilie Bouhsira

**Affiliations:** 1 Department of Parasitology and Parasitic Diseases, University of Agricultural Sciences and Veterinary Medicine of Cluj-Napoca, Calea Mănăștur 3-5, 400372 Cluj-Napoca-Napoca, Romania; 2 Clinical Hospital of Infectious Diseases of Cluj-Napoca, Cluj-Napoca-Napoca, Romania; 3 Department of Parasitology and Zoology, University of Veterinary Medicine, Budapest, Hungary; 4 HUN-REN-UVMB Climate Change: New Blood-Sucking Parasites and Vector-Borne Pathogens Research Group, Budapest, Hungary; 5 Department of Microbiology, University of Agricultural Sciences and Veterinary Medicine of Cluj-Napoca, Calea Mănăștur 3-5, 400372 Cluj-Napoca-Napoca, Romania; 6 InTheres, Université de Toulouse, INRAE, ENVT, Toulouse, France

**Keywords:** Carnivores, Distribution, Diversity, Ectoparasites, Fleas, Siphonaptera, Romania, Wildlife

## Abstract

**Background:**

Fleas are important hematophagous insects, infesting mammals and birds with a worldwide distribution. Fleas of medical importance have been reported from various carnivores worldwide, such as felids, canids, or mustelids. Romania hosts a wide carnivore diversity, but very little is known about flea species that parasitize these animals in Romania. This study aimed to provide a better understanding of the fleas’ diversity and their distribution in a relatively large and diverse number of wild carnivore hosts from Romania.

**Methods:**

From 2013 to 2021, 282 carcasses of wild carnivores from different locations in Romania were collected and examined for the presence of ectoparasites. All collected fleas were morphologically identified using specific keys and descriptions. An analysis of the co-occurrence networks was performed.

**Results:**

A total of 11 flea species were identified: *Pulex irritans* (41.09%), *Paraceras melis* (20.11%), *Ctenocephalides felis* (7.33%), *Ctenocephalides canis* (7.83%), *Monopsyllus sciurorum* (11.11%), *Chaetopsylla trichosa* (21.96%), *Chaetopsylla homoea* (5.5%), *Chaetopsylla tuberculaticeps* (100%), *Chaetopsylla rothschildi* (13.33%), *Chaetopsylla* sp. (14.34%), *Chaetopsylla globiceps* (5.12%), *Echidnophaga gallinacea* (10%). The statistical analyses showed a significant difference between the infestation of *Martes foina* with females being more frequently infected than males (66% versus 33%). *Paraceras melis* infesting *Meles meles* had a significantly higher prevalence in female badgers than in males (× 2 = 7.7977, *P* < 0.01) and higher intensities of infestations in males than in females (*t* = 1.871, *P* < 0.05).

**Conclusions:**

This is the first large-scale study investigating the distribution and diversity of flea species infesting wild carnivores in Romania. Three flea species were identified for the first time in Romania (*E. gallinacea, C. homoea*, and *C. tuberculaticeps*).

**Graphical Abstract:**

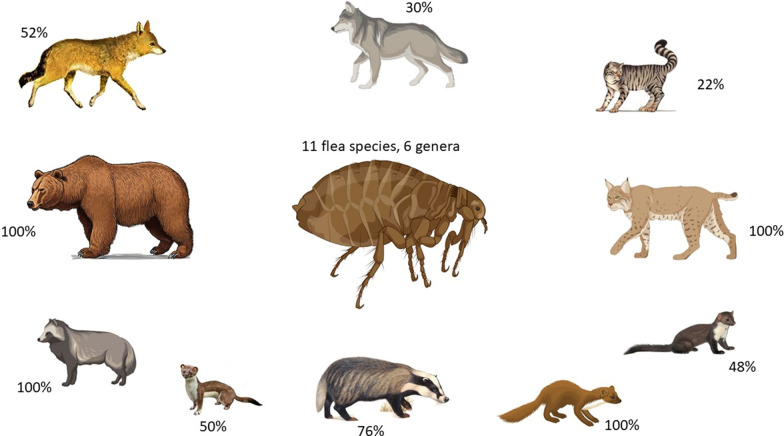

**Supplementary Information:**

The online version contains supplementary material available at 10.1186/s13071-024-06235-3.

## Background

The order Siphonaptera (fleas) is a highly specialized order of obligatory parasitic, holometabolous insects, including 246 genera with more than 2500 species. Fleas are laterally flattened wingless insects, ranging in length from 1 mm to 10 mm. The adults are obligate parasites, with both males and females feeding on the blood of birds and mammals, having a worldwide distribution, including polar regions. They can inhabit an extended range of hosts and habitats, being more diverse in burrowing mammal hosts [1–4]. In contrast to adult stages, immature stages are found in the environment, with larvae feeding on debris from nests. Some of the flea species have great medical importance as vectors of several pathogens, including zoonotic ones, such as *Bartonella* spp., *Coxiella burnetii*, *Rickettsia* spp., *Yersinia pestis,* Myxoma virus, or *Trypanosoma* spp. They can act as intermediate hosts for larval stages of cestodes such as *Dipylidium caninum* and *Hymenolepis* spp. In addition, their bites can cause severe discomfort, associated with dermatological lesions, mainly of allergic nature in pet animals [5–7]. Among the fleas hosts, carnivores represent the second most prevalent group after rodents [8]. Many of the medically important flea species have been reported from various carnivores worldwide, such as felids, canids, or mustelids [4, 9–11]. Moreover, the expansion and urbanization of wild carnivores can contribute to bridging infections and the exchange of parasites with domestic animals [12].

Even though the integrity of the natural ecosystems is generally declining worldwide [13], Romania is still considered a country with a significant coverage of intact habitats, populated by various wild carnivores, such as the brown bear (*Ursus arctos*), grey wolf (*Canis lupus*), golden jackal (*Canis aureus*), red fox (*Vulpes vulpes*), European wildcat (*Felis silvestris*), Eurasian lynx (*Lynx lynx*), and various mustelids [14]. Despite its rich carnivore diversity, very little is known about flea species that parasitize these animals in Romania. A wide diversity of flea species was reported in Romania, mainly from birds and micromammals in a study performed 50 years ago [15]. More recently, two studies investigated the diversity of flea species infesting carnids: one in domestic dogs restricted to Hunedoara county [16] and one in red foxes from the Transylvania region [17]. In red foxes, fleas were morphologically identified as *Ctenocephalides canis*, *Ctenocephalides felis*, *Pulex irritans*, *Chaetopsylla globiceps*, and *Chaetopsylla trichosa*, *Paraceras melis,* and *Ctenophthalmus assimilis* [17], while in domestic dogs, fleas were identified as *Ct. felis*, *Ct. canis*, and *Pu. irritans* [16].

While carnivore assemblage of southeast Europe may be taxonomically diverse [18, 19], generally sympatric carnivores may share a considerable number of the local flea species among them [11, 20–23].

While flea species associated with European carnivores are mostly known, there are no studies on the biotic and abiotic drivers shaping flea assemblages in southeast European carnivores. Apart from host species and life history, environmental conditions and inference competition may affect ectoparasite infestation on any suitable host. While for endoparasites living conditions are stable, ectoparasite densities of vertebrates may be influenced by habitat use and climatic exposure by the host [24]. Moreover, most ectoparasite populations have off-host life stages, usually for an extended time (like all Siphonaptera species do). These periods are spent in the host’s den or in the general environment and may reduce flea populations or the likeliness of subsequent host colonization due to extrinsic factors, such as temperature, humidity, or general lack of hosts. Thus, host and parasite life history, like den-use, general habitat selection (and associated differences in habitat structures), and climate may fundamentally influence local parasitism levels in general, or flea abundance in particular [8, 11, 24].

The present study aimed to describe the flea fauna diversity in a wide and diverse number of wild carnivore hosts along with their distribution in different regions of Romania. These results may contribute to a better understanding of the flea species and their associated flea-borne pathogens that might be shared with domestic animals, as a consequence of the expansion of urbanization.

## Methods

Over 8 years, from 2013 to 2021, 282 carcasses of wild carnivores from different locations in Romania were collected and examined for the presence of ectoparasites. All the carcasses included in this study were collected by hunters or were found as roadkill. The examined hosts were morphologically identified to species level as belonging to several different families, such as Canidae (*n* = 107), Felidae (*n* = 30), Mustelidae (*n* = 144), and Ursidae (*n* = 1). Details about the exact location, date, fleas species, sex, and season of the collection, as well as the number of individual fleas, were all recorded and incorporated into an Excel database. To minimize the risk of post mortem loss of ectoparasites or the incidence of cross-infestation, the carcasses were individually placed in plastic bags as soon as they were hunted or found dead, and the identification details were marked on the bag. Only the carcasses that were maintained at −18 °C were kept for ectoparasite collection and identification to ensure that fleas were conserved properly. A complete necropsy was performed on each of the collected animals. Carcasses were removed from the bags, and a rigorous visual inspection of the wrapping was done to detect any ectoparasite that left the body. Initially, combing of the full body area for 5 min with a fine-tooth flea comb was performed, but without satisfactory results in collecting many ectoparasites, so the fur was further systematically examined starting from the head to the tail, carefully checking all parts of the body. Detected ectoparasites were collected manually, using fine tweezers, and placed in 2 ml labeled tubes with 70% ethanol. Collected fleas were morphologically identified to species or genus level and the DNA was isolated from a maximum of five specimens from each genus per animal species, with the preservation of the exoskeleton for morphological identification to species level as previously described [25]. The remaining exoskeletons were permanently mounted and morphologically identified on the basis of specific keys and descriptions [3, 26–28]. Photographs were taken using an Olympus BX61 microscope attached to a camera and Nikon 80i microscope, and Nikon SMZ1500 stereomicroscope attached to an Axiocam 208 (Zeiss) camera.

The online available Venn diagram tool (http://bioinformatics.psb.ugent.be/webtools/Venn) was used for visualizing the number of shared flea taxa between the most common carnivore species. The general network of host–parasite relationships was graphically represented using a bipartite diagram, using a cumulative index of all individual host–parasite relationships independent of the intensity values [29]. The diagram was created in RStudio (v.2023.03.1), using the “bipartite” package, function “*plotweb*”. To compare parasite prevalence rates, chi-squared (or Fisher’s exact) tests were used. The sample size showed wide variation among the different carnivore host species due to the methodology used (e.g., the use of roadkill specimens). As a low sample size may cause bias (through both zero positives, as well as biased zero interpretation), we tried to reduce it through different groupings used in the analyses (i.e., taxonomic, sex, age, season, and land use). To test the importance of certain biotic factors (host species, sex, age) and abiotic factors (land-use type, season, elevation) on the presence versus absence of fleas or flea abundance or species diversity (number of flea species/host), general linear models (GLM) under the assumption of a binomial distribution (absence/presence), with the built-in *glm* function were used [30]. To test for co-linearity and combined effects of multiple predictors, logistic regression was done, where sampling locality was included as a random effect. Land use and altitude data were extracted for 2 × 2 km cells, for which the centroid was the geo-referenced collection location of the sample. CORINE LandCover (European Environment Agency, http://www.eea.europa.eu/) was used for extracting environmental data, relying on five predictors (Additional file 1: altitude and percent of arable land, grassland, urbanized areas, and forest cover). For the season, the calendar divisions for winter (December–February), spring (March–May), summer (June–August), and autumn (September–November) were used.

Differences were considered significant when *P* < 0.05. The distribution maps based on flea species were generated using ArcMap 10.6.1.

## Results

**Fig. 1:**
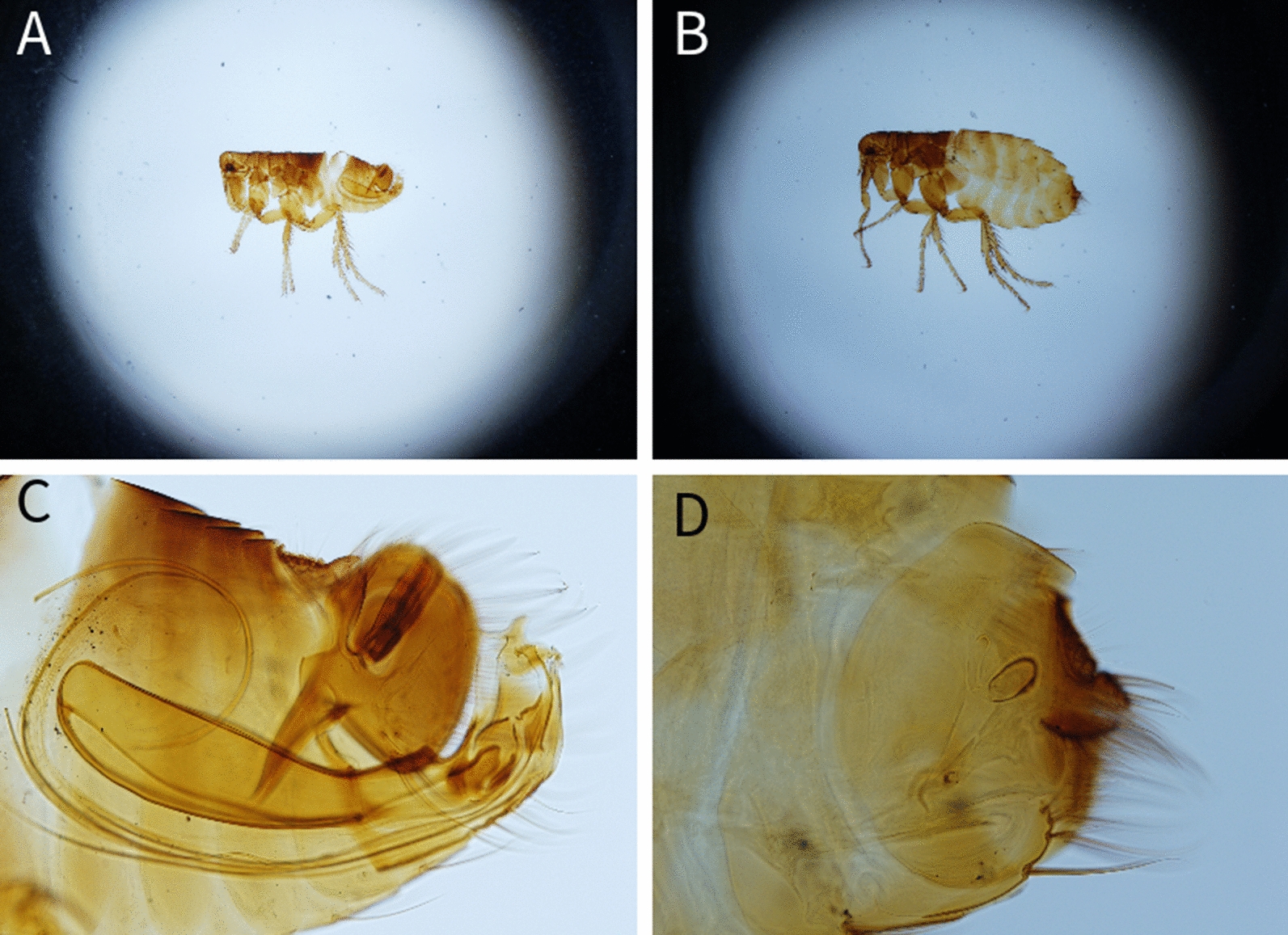
*Chaetopsylla rothschildi*. **A** The general aspect of a male *Ch. rothschildi*; **B** (X1.25). The general aspect of a female *Ch. rothschildi* (X1.25); **C** The magnified aspect of the male genitalia (X10); **D** The magnified aspect of the female genitalia showing the spermatheca

**Fig. 2:**
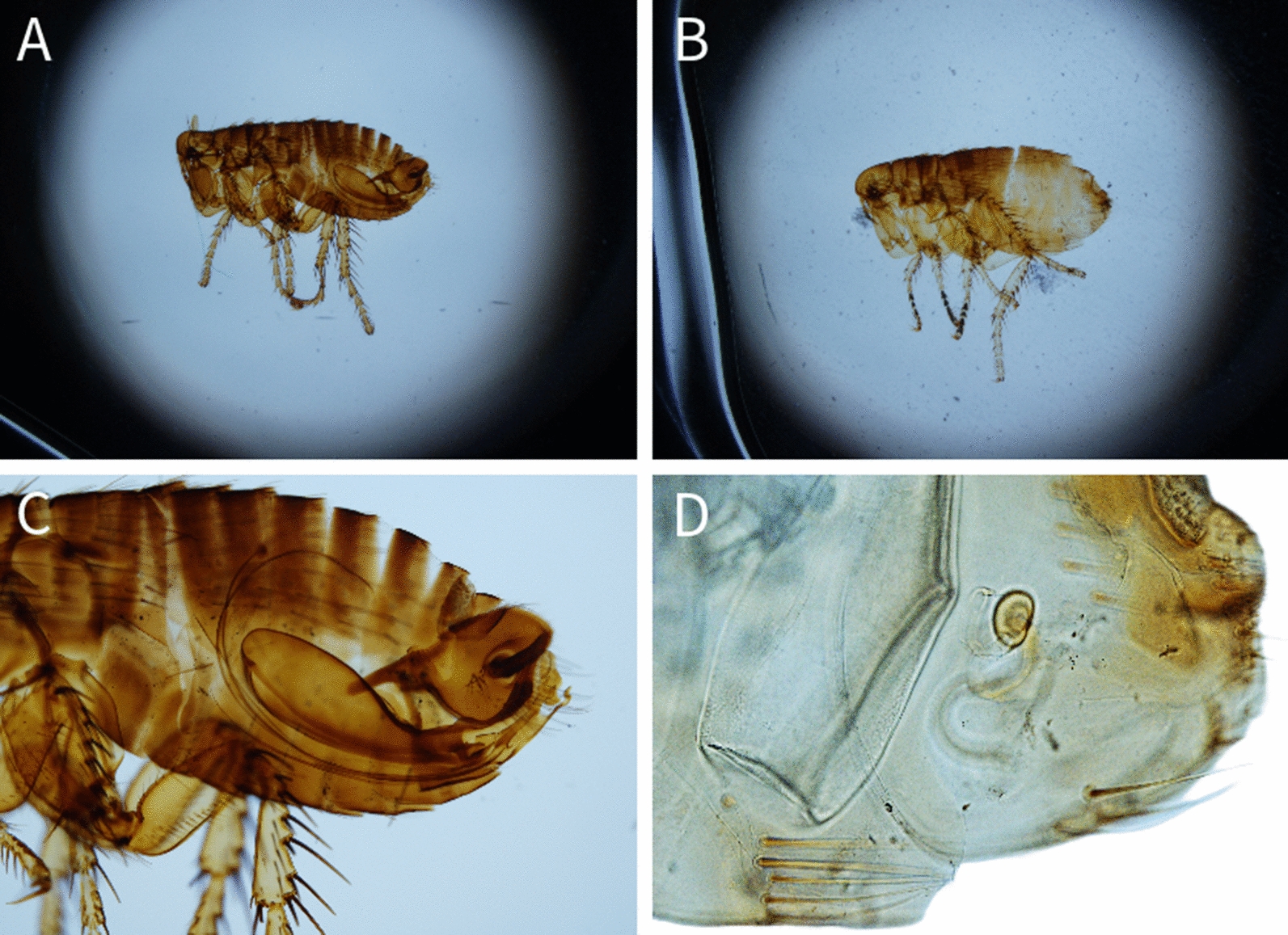
*Chaetopsylla trichosa*. The general aspect of a male *Ch. trichosa*; **B** (X1.25). The general aspect of a female *Ch. trichosa* (X1.25); **C** The magnified aspect of the male genitalia (X10); **D** The magnified aspect of the female genitalia showing the spermatheca (X10)

**Fig. 3:**
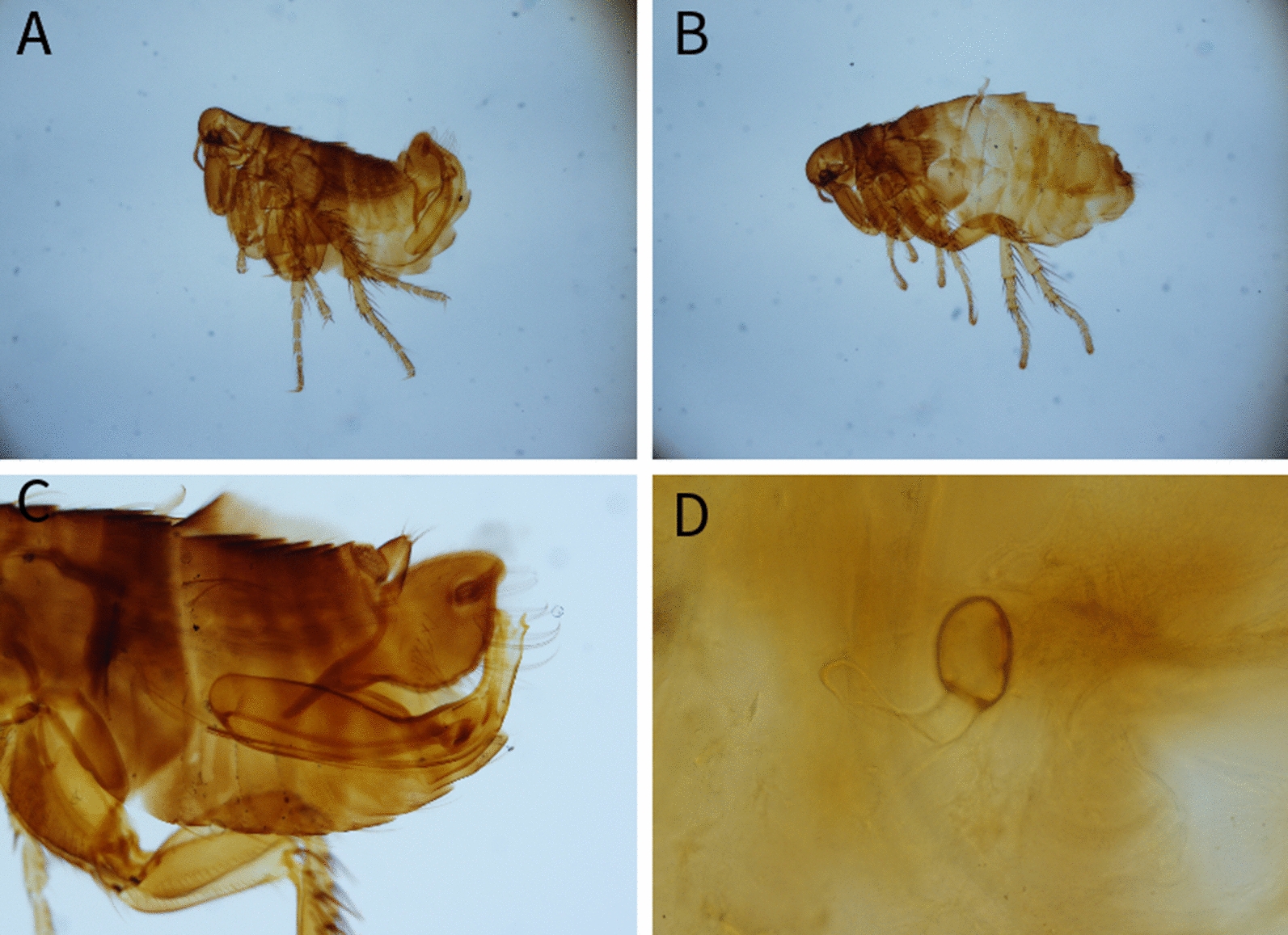
*Chaetopsylla globiceps*. The general aspect of a male *Ch. globiceps*; **B** (X1.25). The general aspect of a female *Ch. globiceps* (X1.25); **C** The magnified aspect of the male genitalia (X10); **D** The magnified aspect of the female spermatheca (X20)

**Fig. 4:**
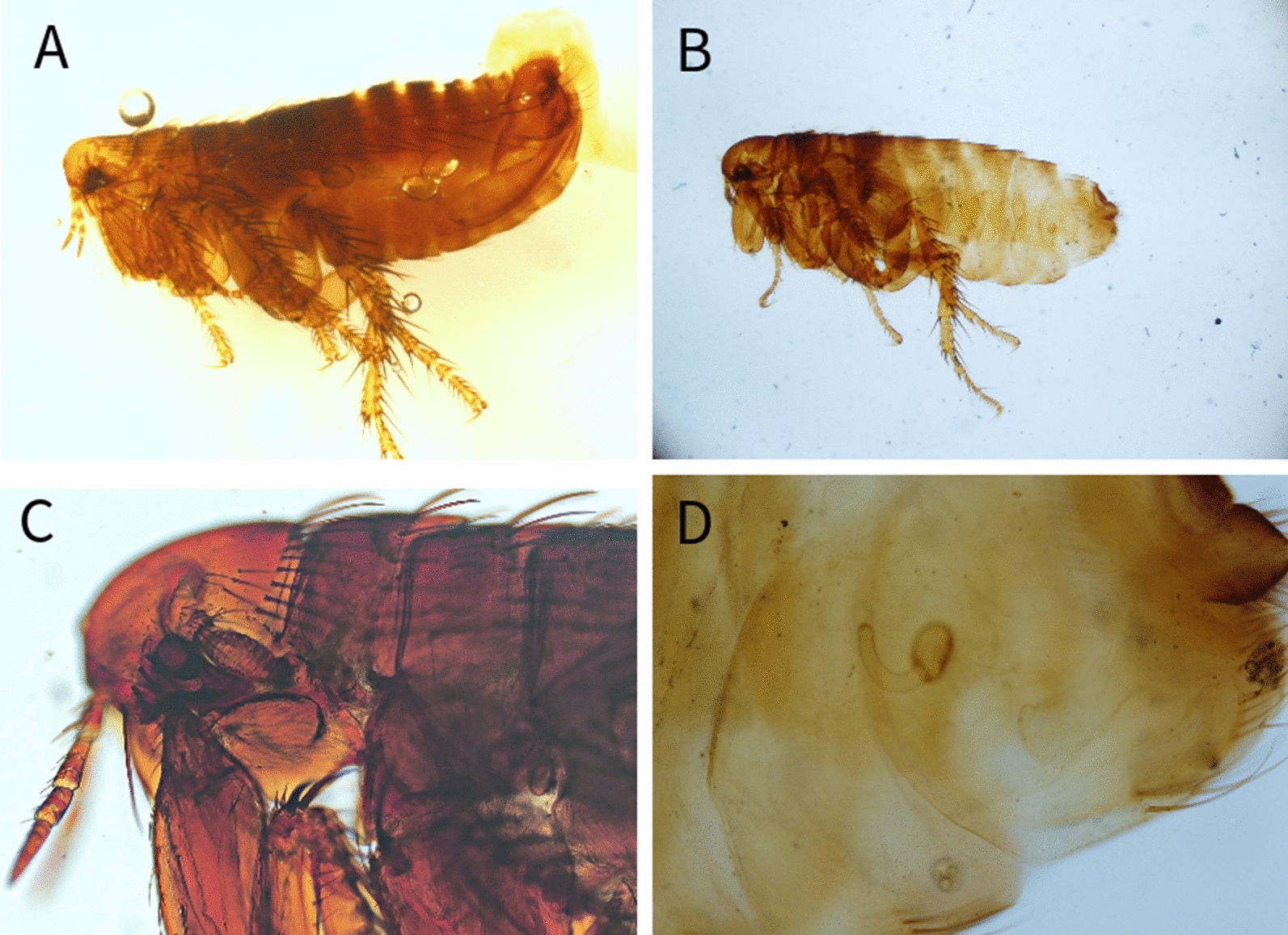
*Chaetopsylla homoea*. The general aspect of a male *Ch. homoea*; **B** (X4). The general aspect of a female *Ch. homoea* (X4); **C** The morphological aspect of the head (X10); **D** The magnified aspect of the female spermatheca (X20)

**Fig. 5:**
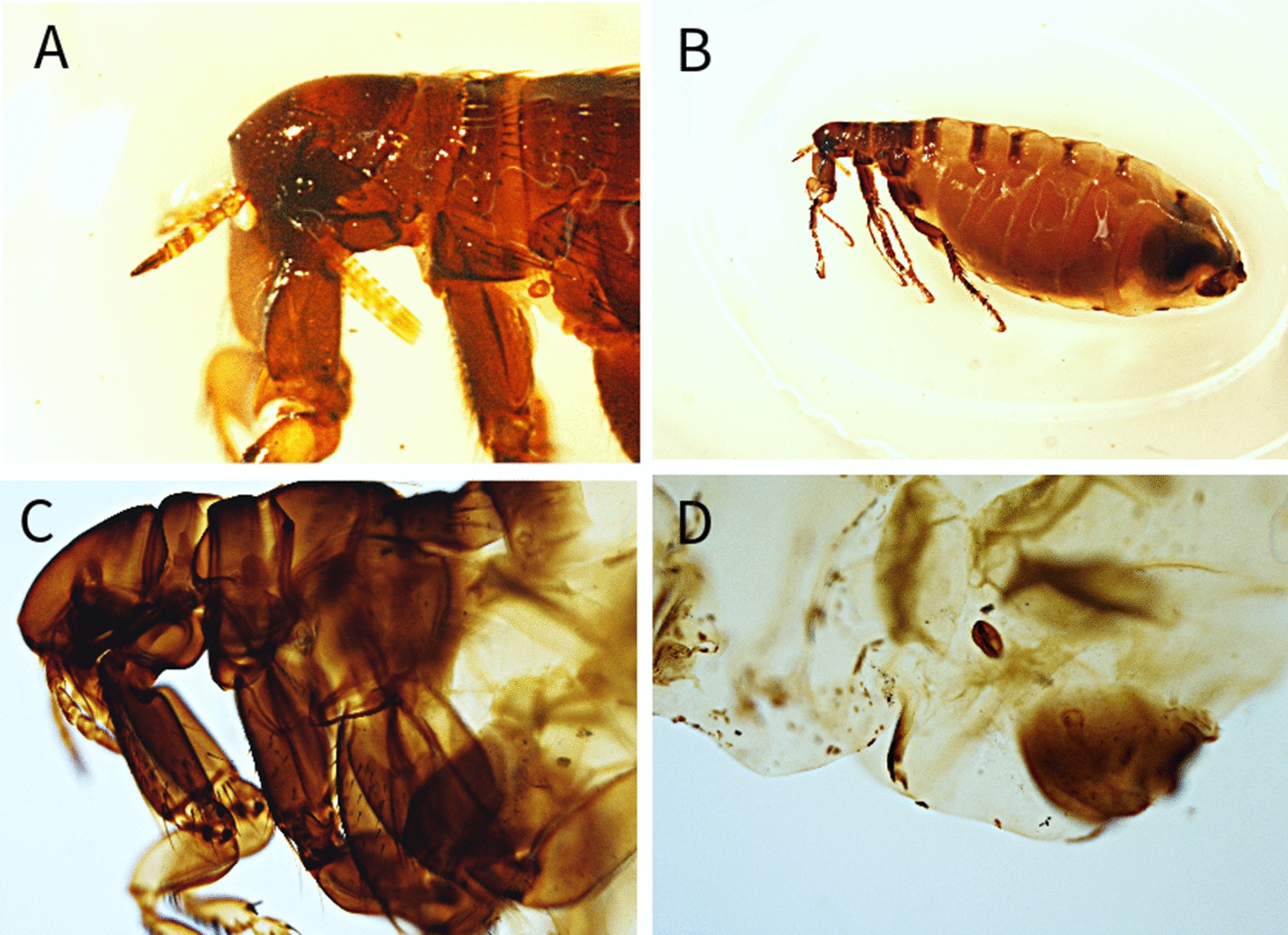
*Chaetopsylla tuberculaticeps*. **A**, **C** The general aspect of the head *Ch. tuberculaticeps* (X4); **B** The general aspect of a female *Ch. tuberculaticeps* (X1.25); **D** The magnified aspect of the female spermatheca (X10)

**Fig. 6:**
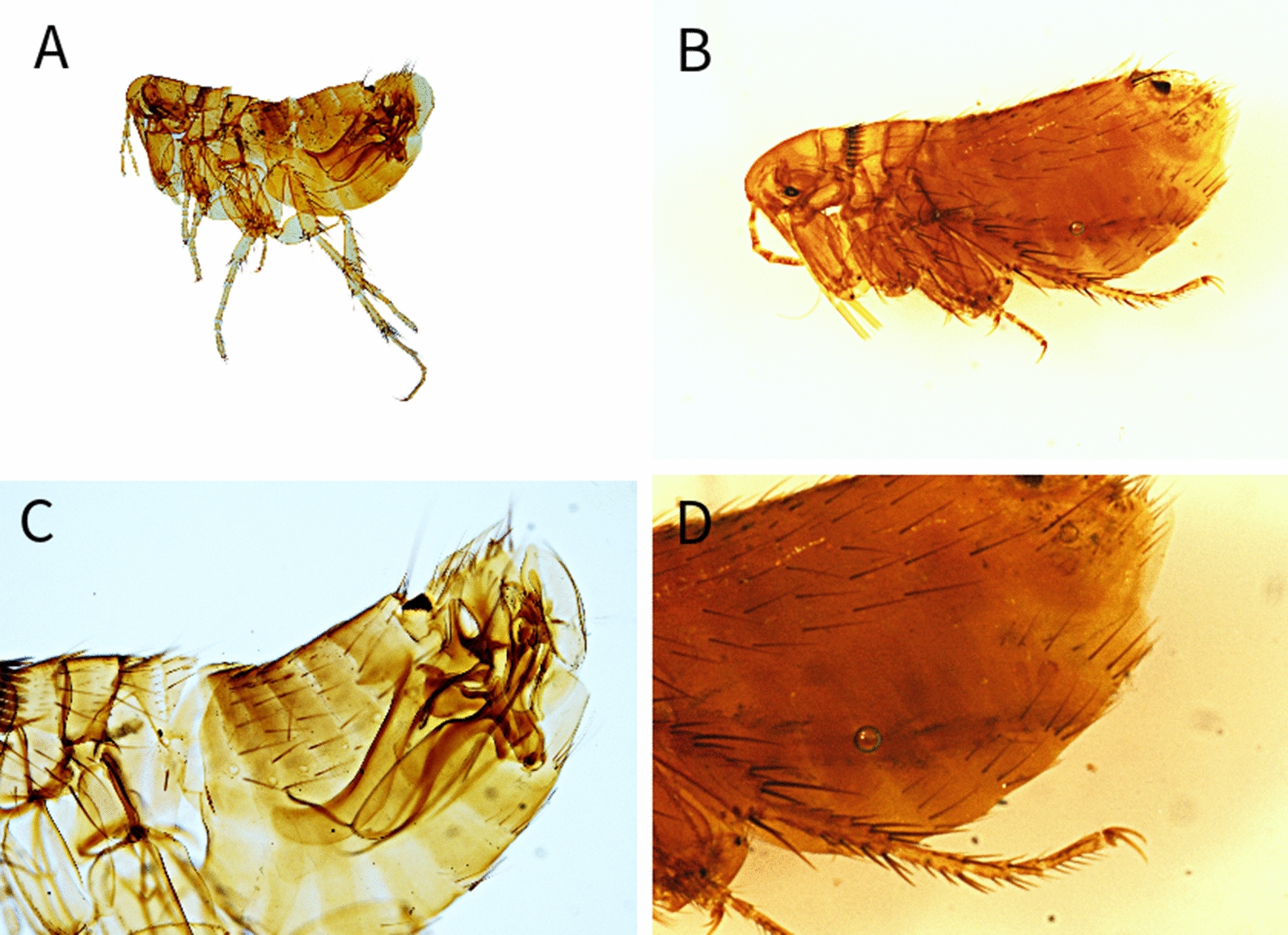
*Paraceras melis*. **A** The general aspect of a male *Pa. melis* (X4); **B** The general aspect of a female *Pa. melis* (X4); **C** The magnified aspect of the male genitalia (X10); **D** The magnified aspect of the female genitalia showing the spermatheca (X10)

**Fig. 7:**
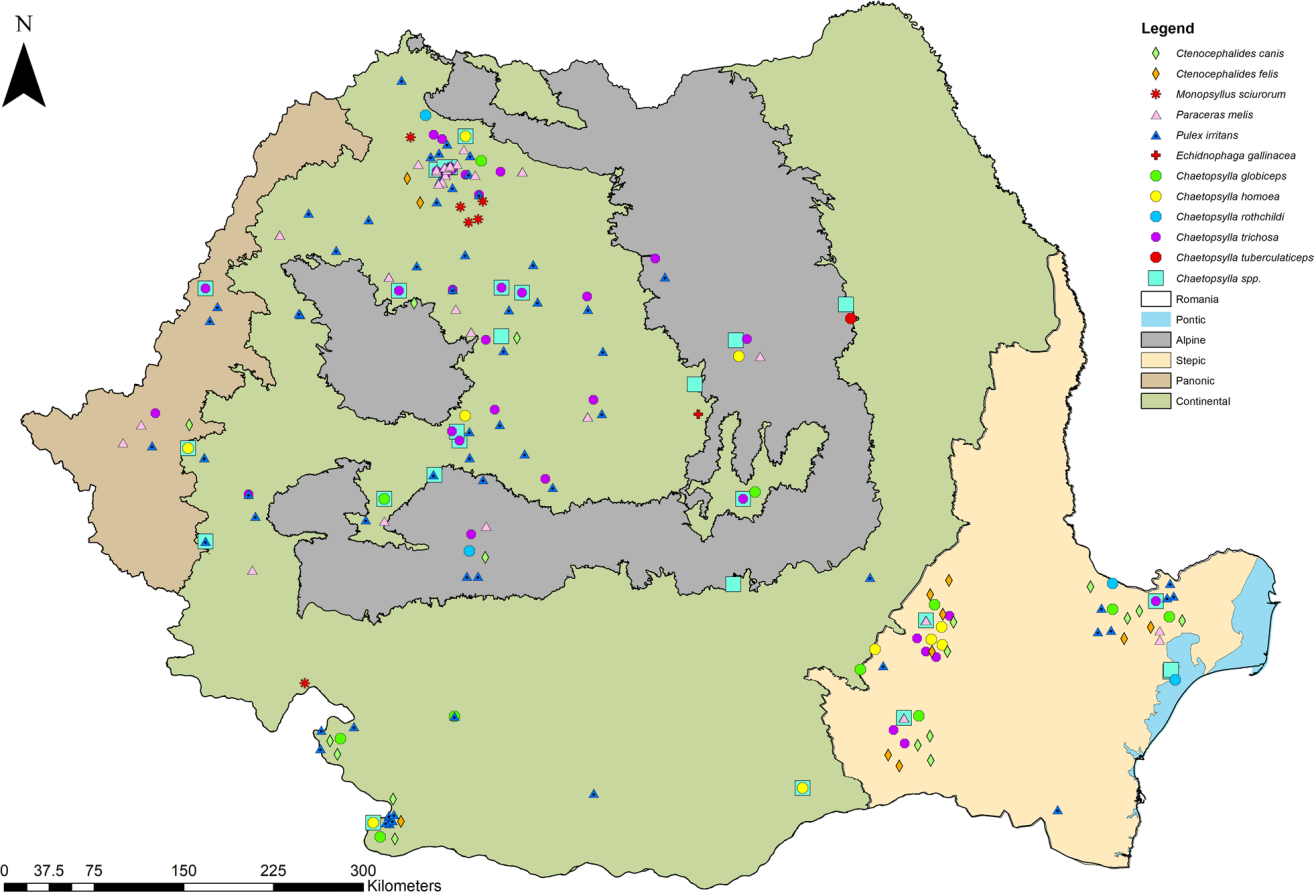
Map showing the geographical distribution of identified fleas correlated to the bioregions

Among the examined carnivores (Table 1), a total of 11 different flea species belonging to 6 genera were identified (Table 2) (Figs. 1, 2, 3, 4, 5, 6). The most prevalent flea species was *Pulex irritans* (41.09%, 106/258) identified in eight different host species [golden jackal, grey wolf, European wildcat, Eurasian lynx, Eurasian badger (*Meles meles*), raccoon dog (*Nyctereutes procyonoides*), beech marten (*Martes foina*), and European pine marten (*Martes martes*)], followed by *Chaetopsylla trichosa* (21.96%, 47/214) in three different host species (golden jackals, European wildcat, and Eurasian badger) and *Paraceras melis* (20.11%, 38/189) in three different host species [golden jackals, Eurasian badgers, and least weasel (*Mustela nivalis*)]. Due to the long-term preservation period and degradation or evaporation of the ethanol and dryness of fleas, some specimens of *Chaetopsylla* (14.24%, 37/258) were identified only to the genus level. *Chaetopsylla tuberculaticeps* (100%, 1/1) in one brown bear, *Ch. homoea* (5.5%, 12/218) in five different hosts (golden jackal, beech marten, European pine marten, raccoon dog, and Eurasian badger) and *Echidnophaga gallinacea* (10%, 1/10) in a single grey wolf were identified. The identified fleas and their distribution in correlation with the bioregions are shown in Fig. 7.

**Table 1:** Animal hosts infested by fleas and prevalence per host

Species	No. examined hosts	No. hosts with fleas	Prevalence (%)
Golden jackal (*Canis aureus*)	96	50	52.08
Grey wolf (*Canis lupus*)	10	3	30
European wildcat (*Felis silvestris*)	27	6	22.22
Eurasian lynx (*Lynx lynx*)	3	3	100
Beech marten (*Martes foina*)	27	13	48.15
European pine marten (*Martes martes*)	3	3	100
Eurasian badger (*Meles meles*)	91	69	75.82
Least weasel (*Mustela nivalis*)	2	1	50
Raccoon dog (*Nyctereutes procyonoides*)	1	1	100
Brown bear (*Ursus arctos*)	1	1	100

**Table 2:** Prevalence and intensity of infestation for identified fleas

Flea species	No. host species	Examined hosts	Infected hosts	Prevalence (%)	Intensity	Flea type	Host group
*Pulex irritans*	8	258	106	41.09	7.83	G	Carnivora
*Paraceras melis*	3	189	38	20.11	4.39	S	*Meles meles*
*Ctenocephalus canis*	4	217	17	7.83	2.18	G	Carnivora
*Ctenocephalus felis*	3	150	11	7.33	1.36	G	Carnivora
*Monopsyllus sciurorum*	2	54	6	11.11	1.67	S	Dormice/squirrel
*Chaetopsylla globiceps*	4	215	11	5.12	1.82	G	Carnivora
*Chaetopsylla homoea*	5	218	12	5.5	2.25	G	Carnivora
*Chaetopsylla rothschildi*	2	30	4	13.33	3	S	*Martes* spp.
*Chaetopsylla trichosa*	3	214	47	21.96	4.49	S	Carnivora
*Chaetopsylla tuberculaticeps*	1	1	1	100	NA	S	*Ursus arctos*
*Chaetopsylla* sp.	8	258	37	14.34	4.3		
*Echidnophaga gallinacea*	1	10	1	10	NA	G	Many

*G* generalist (wide palette of carnivoran species used)

*S* specialist (single or small number of carnivora species used)

The statistical analyses showed a significant difference in the infestation of *Martes foina*, with females being more frequently infected than males (66% versus 33%). On the contrary, male martens had higher intensities of infestation than females (Additional file 1, *t* = 2.6372, *P* = 0.01). On the basis of flea species, a significant difference was detected for *Pa. melis* infesting *Me. meles*, with a significantly higher prevalence in female badgers than in males (*χ*^2^ = 7.7977, *P* < 0.01) and higher intensities of infestations in males than in females (Additional file 1, *t* = 1.871, *P* < 0.05). *Chaetopsylla trichosa* infesting *Me. meles* was significantly more prevalent in males than females (*χ*^2^ = 1.6319, *P* < 0.05). The GLM showed that, overall, the only factor that influenced the infestation by fleas was the sex of the host (*P* = 0.0555), while all other analyzed parameters showed no correlations (Additional file 1). A high number of female *Ma. foina* were infested by *Pu. irritans* compared with males, in which this flea species was not identified at all. Although very few *Ca. aureus* were positive for *Ct. felis*, male hosts were more commonly infested than females, while the intensity of infestation was higher in females. *Chaetopsylla homoea* was more prevalent in female *Ca. aureus*, but few hosts were infested by this flea species.

**Fig. 8:**
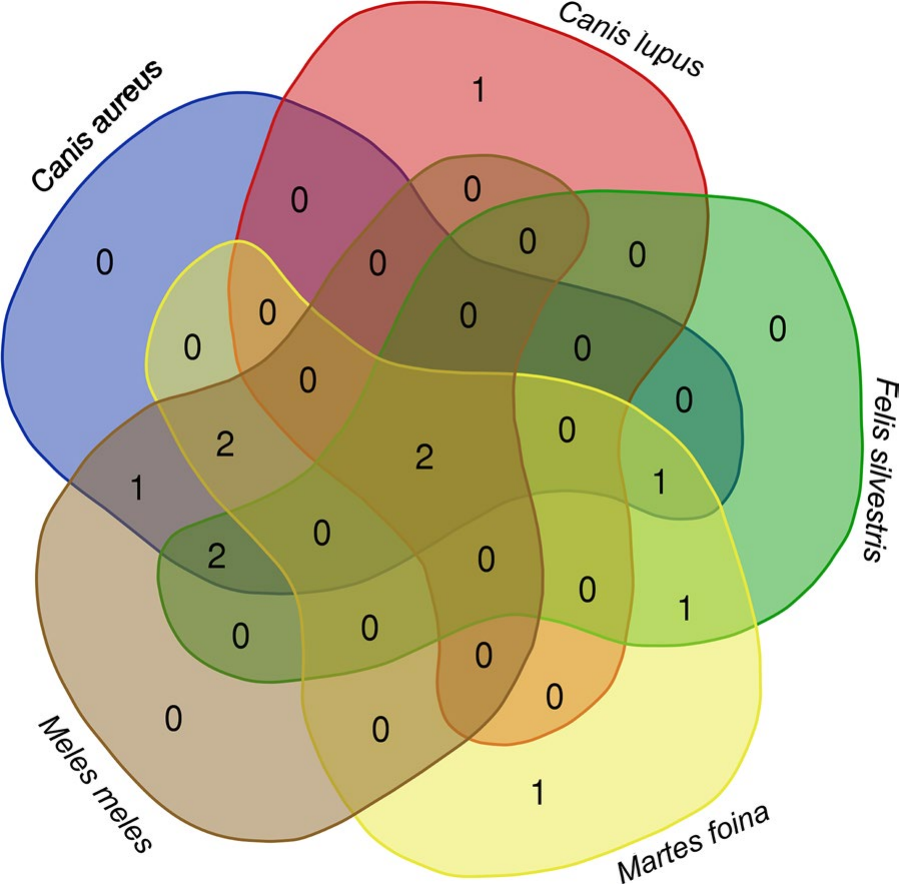
Venn diagrams of co-occurrence networks of flea species in different carnivore species. The Venn diagrams show the number of central nodes using betweenness centrality (i.e., fleas that are common or unique among the five host species)

**Fig. 9:**
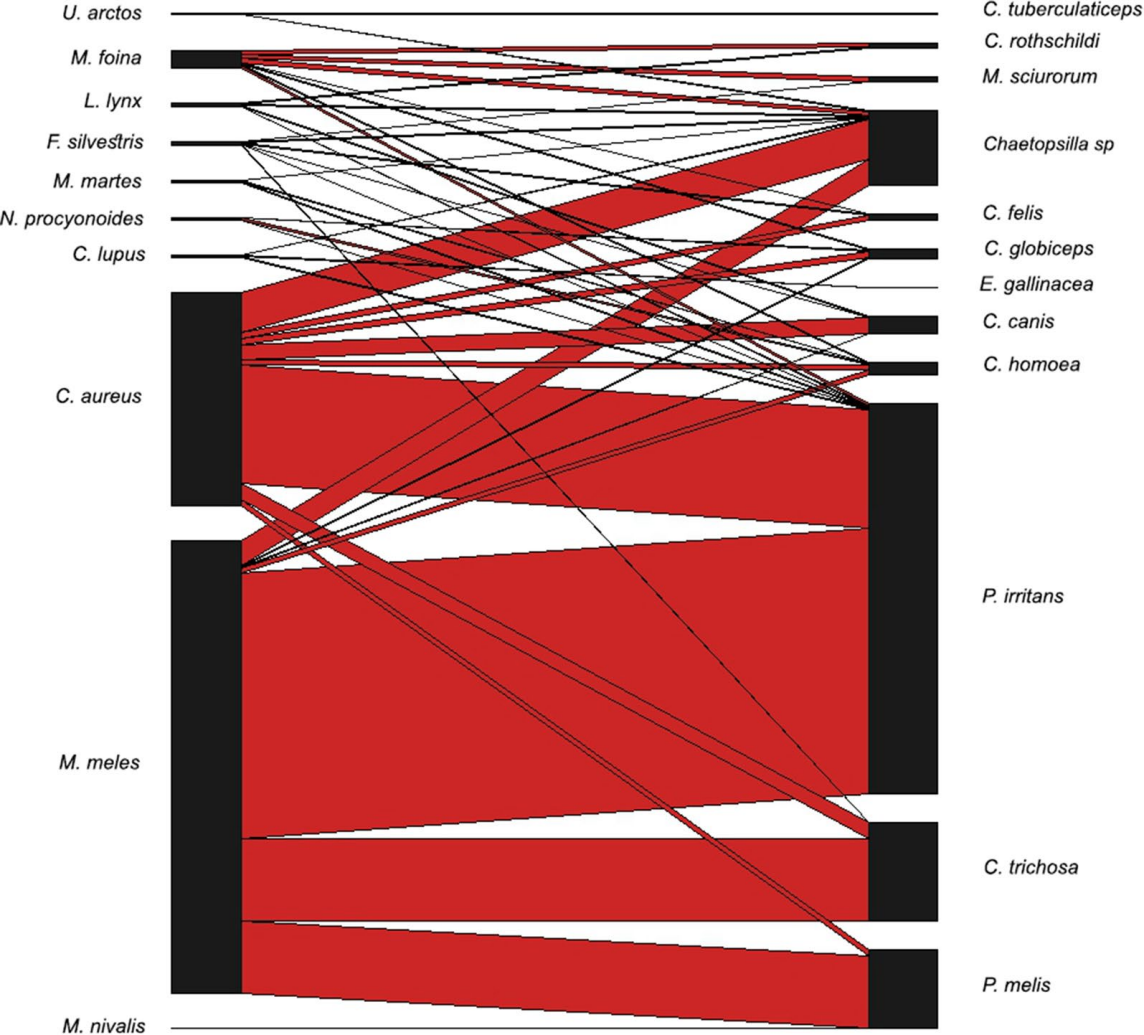
Bipartite representation of the parasite network of carnivores and flea species in Romania

None of the selected biotic (host species, sex, or age) or abiotic (season, altitude, climate) predictors were crucial in determining overall flea intensity; however, flea species diversity was negatively correlated with host sex (males had significantly lower diversity). The core host–parasite network shows two shared species (*Pu. irritans* and *Chaetopsylla* spp.) among all hosts, which are also the most important components of the host–parasite networks (85%) among those carnivores that had enough sample size (Fig. 8). Other hosts showed lower overlaps in shared parasite species and a negative correlation between the intensity of infestation and the number of flea species, meaning that in higher intensities, fewer flea species are observed. The detailed host–parasite relations of all flea species (including prevalence and intensity of infestation) are presented in Table 2, while the distribution of host–parasites relationships is shown in Fig. 9.

## Discussion

A high number of wild carnivores from Romania are hosts for a variety of flea species. In golden jackals and badgers, the flea diversity and mean intensity of infestation were higher than in other hosts. These are burrow-dwelling species and are more likely to be infested. The higher flea diversity in golden jackals and badgers could be also influenced by the higher number of examined hosts, as parasite occurrence shows cumulative curves [31]. Wildcats and beech martens were the only two hosts infested by *Monopsyllus sciurorum*, a specialist flea of dormice and squirrels, highlighting the targeted predation on these rodents by wildcats and martens [32–34]. An interesting finding was *Echidnophaga gallinacea*, also called the “sticktight flea” in a grey wolf. *E. gallinacea* is a sedentary flea that attaches around the head and wattle of domestic and wild birds, but it was also identified in rats, domestic dogs, and wild canids, cats, horses, pigs, and even humans [11, 35]. Its finding for the first time in Romania in a single animal might be related to the feeding behavior of the grey wolves. Grey wolves are opportunistic foragers, and more than 30% of their diet is based on birds, livestock, and rodent carrions [36, 37]. Considering the low number of wolves examined and their feeding habits, the identification of *E. gallinacea* in this host is not surprising, and the previous lack of reports in the country may be related to the lack of studies rather than a real absence of the flea. *Chaetopsylla tuberculaticeps*, another flea identified for the first time in Romania in brown bears, was previously described in grizzly bears (*Ursus arctos horribilis*) in North America [38] and in brown bears in Russia, Japan, Norway, and northern Italy [28, 39]. Besides bears, *Ch. tuberculaticeps* was also identified from a man who had contact with a bear den and from a domestic dog that stayed in a garage with a dead *Ursus americanus* [39]. Two subspecies of *Ch. tuberculaticeps* were described, namely *Ch. t. ursi* in America and *Ch. t. tuberculaticeps* in Eurasia. The main morphological differences between the two subspecies include the antennal club of male fleas being longer and almost parallel sides and the anterior apical angle of phallosome with a rectangular form for *Ch. t. ursi.* However, there are no clear differences in female fleas, and in the present case, only two female specimens were detected and identified [39]. In addition, there are no sequences available in GenBank for molecular identification of the subspecies, but considering the geographical location, it can be assumed that Romanian samples belong to *Ch. t. tuberculaticeps*. The third new species for Romania’s flea fauna was *Ch. homoea,* which is considered abundant in alpine areas and was mostly identified in mustelids. The species was reported in dogs from Kyrgyzstan, in *Mu. erminea* in Kazakstan, in a canid host in the Altai Republic, and a domestic dog in Tibet [26]. In Europe, it was identified in domestic dogs and foxes in Switzerland [27, 28], in mustelids and red foxes in France [40, 33], and mustelids from Italy [41]. In contrast to the general knowledge regarding *Ch. homoea*, our study identified it only in four mustelid species, while it is more common among canid hosts (seven golden jackals and one raccoon dog). Additionally, most records came from lowland areas of continental and steppic bioregions, with a single record (one badger) collected from the alpine area (see distribution map in Fig. 7). An adaptation of this flea species to warmer climates may also be considered.

The sex-related differences in flea infestation in the case of *Martes foina* may be related to differences in behavior between the sexes. Female stone martens hunt and feed more frequently than males, which makes them much more exposed to flea infestation, or they can be more exposed during the reproduction act, as males have contact with several females [42]. In addition, females show much higher fidelity and den-use, especially in the young-rearing period [43, 44], thus favoring higher chances for flea encounters. Animals’ sex seems to be the only risk factor related to flea infestation. This assessment is also supported by the finding that *Pa. melis* is more common on female badgers, *Pu. irritans* is identified only on female *Ma. foina,*, and *Ch. homoea* on female golden jackals. Interestingly, in contrast to the distribution of other species, the cat flea (*Ct. felis*) was more prevalent in male *Ca. aureus* than on females. The less commonly infested animals (lower prevalences) based on their sex within an animal species proved to be more intensely infested, meaning that they spend more time around nests or burrows and maybe do not often have contact with other potential hosts for the transmission of the fleas. However, we acknowledge the major weakness of this study, which is the examination of carcasses mostly collected from car accidents, and that some fleas most likely left the host before the carcasses were collected. This fact may have influenced the results differently. To the best of our knowledge, there are no data about which flea species react first to the fall of body temperature of the host to predict species loss.

## Conclusions

This is the first large-scale study investigating the distribution and diversity of flea species infesting wild carnivores in Romania. Three flea species were identified for the first time in Romania (*E. gallinacea, Ch. homoea*, and *Ch. tuberculaticeps*).

### Supplementary Information

**Additional file 1. **Animals. The sheet contains details about animal location, sex, age, and the identified flea species; Environmental predictors. Correspondence between CORINE LandCover categories and land use types used in this study; Host_sel. This sheet shows the intensity and prevalences of all flea species and their type; Flea host report. The sheet shows the animal species and their prevalences of infestation on the basis of their sex; Flea species results. This sheet shows a correlation between the animal species and the fleas; GLm results. This sheet shows the statistical results of the animal hosts correlated with the season and age; *Pulex irritans*. This sheet represents all animals infested with *Pu. irritans* and differences based on their sex;* Pa. melis* This sheet represents all animals infested with *Pa. melis* and differences based on their sex;* Ct. felis*. This sheet represents all animals infested with *Ct. felis* and differences based on their sex;* Ct. canis*. This sheet represents all animals infested with *Ct. canis* and differences based on their sex;* M. sciurorum*. This sheet represents all animals infested with *M. sciurorum* and differences based on their sex;* Ch. trichosa.* This sheet represents all animals infested with *Ch. trichosa* and differences based on their sex;* Ch. homoea*. This sheet represents all animals infested with *Ch. homoea* and differences based on their sex;* Ch. tuberculaticeps*. This sheet represents all animals infested with *Ch. tuberculaticeps* and differences based on their sex;* Ch. rothchildi*. This sheet represents all animals infested with *Ch. rothchildi* and differences based on their sex;* Ch. globiceps.* This sheet represents all animals infested with *Ch. globiceps* and differences based on their sex;* E. gallinacea*. This sheet represents all animals infested with *E. gallinacea* and differences based on their sex.

## Data Availability

All data generated and analyzed during this study are included in this published article [and its Additional file 1]. The exoskeletons are available on appropriate request from the first author and are kept in the collection in Cluj-Napoca.

## References

[1] Marshall AG (1981). The ecology of ectoparastic insects.

[2] Pilgrim RLC (1991). Fleas. N. Z. Entomol.

[3] Whitaker AP. Fleas: Siphonaptera. Field Studies Council. 2007

[4] Márquez FJ, Millán J, Rodriguez-Liebana JJ, Garcia-Egea I, Muniain MA (2009). Detection and identification of *Bartonella* sp. in fleas from carnivorous mammals in Andalusia, Spain. Med Vet Entomol.

[5] Dobler G, Pfeffer M (2011). Fleas as parasites of the family Canidae. Parasit Vectors.

[6] Durden LA, Traub R, Mullen GR, Durden LA (2002). Fleas (Siphonaptera). Medical and veterinary entomology.

[7] Bitam I, Dittmar K, Parola P, Whiting MF, Raoult D (2010). Fleas and flea-borne diseases. Int J Infect Dis.

[8] Krasnov BR (2008). Functional and evolutionary ecology of fleas: a model for ecological parasitology.

[9] McGee BK, Butler MJ, Pence DB, Alexander JL, Nissen JB, Ballard WB, Nicholson KL (2006). Possible vector dissemination by swift foxes following a plague epizootic in black-tailed prairie dogs in northwestern Texas. J Wild Dis.

[10] Lizundia R, Newman C, Buesching CD, Ngugi D, Blake D, Sin YW, McKeever D (2011). Evidence for a role of the host-specific flea (* Paraceras melis*) in the transmission of* Trypanosoma*(* Megatrypanum*)* pestanai* to the European badger. PLoS ONE.

[11] López-Pérez AM, Gage K, Rubio AV, Montenieri J, Orozco L, Suzan G (2018). Drivers of flea (Siphonaptera) community structure in sympatric wild carnivores in northwestern Mexico. J Vector Ecol.

[12] Feranec J, Jaffrain G, Soukup T, Hazeu GW (2010). Determining changes and flows in European landscapes 1990–2000 using CORINE land cover data. Appl Geogr.

[13] https://wilderness-society.org/romanian-conflict-and-coexistence-with-large-carnivores. Accessed 4 Apr 2023

[14] Polley L (2005). Navigating parasite webs and parasite flow: emerging and re-emerging parasitic zoonoses of wildlife origin. Int J Parasitol.

[15] Suciu M. Catalogue of the Siphonaptera from Romania. Studii şi Comunicări, Muzeul de Ştiinţele Naturii Bacău, Bacău. 1973; 47–72.

[16] Morariu S, Dărăbuș G, Suici T, Rotaru V, Glogovețan L, Imre M (2019). Species of fleas present in Hunedoara County and their role as vectors. Revista Română de Medicină Veterinară.

[17] Foley P, Foley J, Sándor AD, Ionică AM, Matei IA, D’Amico G, Mihalca AD (2017). Diversity of flea (Siphonaptera) parasites on red foxes (*Vulpes vulpes*) in Romania. J Med Entomol.

[18] Baquero RA, Tellería JL (2001). Species richness, rarity and endemicity of European mammals: a biogeographical approach. Biodivers Conserv.

[19] Cazacu C, Adamescu MC, Ionescu O, Ionescu G, Jurj R, Popa M, Cotovelea A (2014). Mapping trends of large and medium size carnivores of conservation interest in Romania. Ann Forest Res.

[20] Matthee S, Van Der Mescht L, Wilson B, Lamberski N (2011). Flea diversity on small carnivores in the Northern Cape Province, South Africa. Afr Zool.

[21] Medvedev SG, Seryodkin IV (2019). Fleas (Siphonaptera) of carnivores (Mammalia, Carnivora) of the Russian Far East. Entomol Rev.

[22] Millán J, Ruiz-Fons F, Márquez FJ, Viota M, López-Bao JV, Paz M-M (2007). Ectoparasites of the endangered Iberian lynx *Lynx pardinus* and sympatric wild and domestic carnivores in Spain. Med Vet Entomol.

[23] Munkhzul T, Murdoch JD, Reading RP (2018). Ectoparasites on meso-carnivores in the desert-steppe of Mongolia. Mongol J Biol Sci.

[24] Morand S, Krasnov BR, Poulin R (2007). Micromammals and macroparasites: from evolutionary ecology to management.

[25] Deak G, Safarov A, Xie XC, Wang R, Mihalca AD, Šlapeta J (2022). Fleas from the Silk Road in Central Asia: identification of *Ctenocephalides canis* and *Ctenocephalides orientis* on owned dogs in Uzbekistan using molecular identification and geometric morphometrics. Parasit Vectors.

[26] Hopkins GHE, Rothschild M. An illustrated catalogue of the Rothschild collection of fleas (Siphonaptera) in the British Museum (Natural History) with keys and short descriptions for the identification of families, genera, species and subspecies of the order. Volume I. Tungidae and Pulicidae. 1953.

[27] Hopkins GHE, Rothschild M. An illustrated catalogue of the Rothschild collection of fleas (Siphonaptera) in the British Museum (Natural History), volume II: Coptopsyllidaw, Vermipsyllidae; Stephanocircidae; Ischnopsyllidae, Hypsophthalmidae and Xiphiopsyllidae Hardcover – January 1, 1956.

[28] Beaucournu JC, Launay H. Les puces (Siphonaptera) de France et du Bassin méditerranéen occidental: par Jean-Claude Beaucournu et Henri Launay. Lechevalier. 1990.

[29] Dormann CF, Gruber B, Fruend J (2008). Introducing the bipartite package: analysing ecological networks. R News.

[30] R Core team. R: A language and environment for statistical computing. Vienna: R Foundation, Vienna, Austria; 2018.

[31] Botero-Cañola S, Gardner SL (2023). Tapping into natural history collections to assess latitudinal gradients of parasite diversity. Parasitol.

[32] Lurz PW, Gurnell J, Magris L (2005). Sciurus vulgaris. Mamm Species.

[33] Apostolico F, Vercillo F, La Porta G, Ragni B (2016). Long-term changes in diet and trophic niche of the European wildcat (*Felis silvestris silvestris*) in Italy. Mamm Res.

[34] Twining JP, Montgomery WI, Tosh DG (2020). The dynamics of pine marten predation on red and grey squirrels. Mamm Biol.

[35] Little SE (2021). Fleas and lice. In Greene’s infectious diseases of the dog and cat.

[36] Newsome TM, Boitani L, Chapron G, Ciucci P, Dickman CR, Dellinger JA, Ripple WJ (2016). Food habits of the world’s grey wolves. Mamm Rev.

[37] Newsome TM, Fleming PJ, Dickman CR, Doherty TS, Ripple WJ, Ritchie EG, Wirsing AJ (2017). Making a new dog?. Bioscience.

[38] Rogers LL, Rogers SM (1976). Parasites of bears: a review. Bears Biol Manag.

[39] Haas GE, Wilson N, Zarnke RL, Barrett RE, Rumfelt T (1982). Siphonaptera from mammals in Alaska. Supplement III. Western Alaska. Can J Zool.

[40] Beaucournu JC (1973). Notes sur les siphonaptères parasites de carnivores en France. Ann Parasitol Hum Comp.

[41] Biocca E, Balbo T, Costantini R (1975). Observations of fleas of small mammals at the Gran Paradiso National Park (Italian Occidental Alps). Parassitologia.

[42] Bakaloudis DE, Vlachos CG, Papakosta MA, Bontzorlos VA, Chatzinikos EN (2012). Diet composition and feeding strategies of the stone marten (* Martes foina*) in a typical Mediterranean ecosystem. Sci World J.

[43] Zalewski A (1997). Patterns of resting site use by pine marten *Martes martes* in Białowieża National Park (Poland). Acta Theriol.

[44] Herr J, Schley L, Engel E, Roper TJ (2010). Den preferences and denning behaviour in urban stone martens (*Martes foina*). Mammal Biol.

